# Informal financial education and consumer financial capability: The mediating role of financial knowledge

**DOI:** 10.3389/fpsyg.2022.1042085

**Published:** 2022-11-04

**Authors:** Fuzhong Chen, Xiuli Lu, Wenting Wang

**Affiliations:** ^1^The Academy of China Open Economy Studies, University of International Business and Economics, Beijing, China; ^2^School of International Trade and Economics, University of International Business and Economics, Beijing, China; ^3^Department of Big Data and Internet, Shenzhen Technology University, Shenzhen, China

**Keywords:** informal financial education, financial capability, mediating effect, financial knowledge, ordered probit regression

## Abstract

With the development of the economy, family wealth continues to accumulate, and more and more consumers participate in financial management affairs. As an important way to improve financial knowledge, informal financial education is vital to consumer financial capability. Utilizing data from the 2012, 2015, and 2018 US National Financial Capability Study and the approaches of ordinary least squares and ordered probit regression are employed to produce more accurate estimates. Meanwhile, the study also explores the mediating effects of financial knowledge between informal financial education and consumer financial capability. The results show that informal financial education has a positive effect on the improvement of consumer financial capability. Besides, financial knowledge partially mediates the nexus between informal financial education and consumer financial capability. Therefore, policymakers are encouraged to formulate measures to promote financial education programs not only in schools and universities but also in workplaces or communities. Companies also should offer more opportunities for their employees to receive financial education and further enhance their financial capability. Consumers should be aware of the importance of financial education and actively learn financial knowledge to improve financial capability and further enhance financial satisfaction.

## Introduction

With the advancement of economic globalization, consumers are living in a gradually financialized world. The improvement of China’s financial market has had a profound impact on consumers’ daily lives. Financial knowledge and financial skills have played increasingly prominent roles in financial decision-making (Greenberg and Hershfield, 2019). Previous studies have shown that the lack of financial knowledge can lead to undesirable financial decision-making behaviors, which will negatively affect the wellbeing of individuals and communities (Collins, 2012). With the current inadequacy in China’s financial market, investment risks continue to increase, social background risk exerts a vital impact on household portfolios, which may lead to the investment of household assets turning to safer and less risky fields (Brown et al., 2021). Therefore, more and more consumers have begun to consciously learn certain financial knowledge, to better contact and integrate into the modern financial market and improve the level of consumer wellbeing by obtaining investment income, which also makes the demand for financial education continues to augment. In addition, there is evidence suggesting that financial education courses have a significantly positive impact on the financial knowledge and financial behavior of low-income groups (Kaiser et al., 2022).

**Figure 1:**
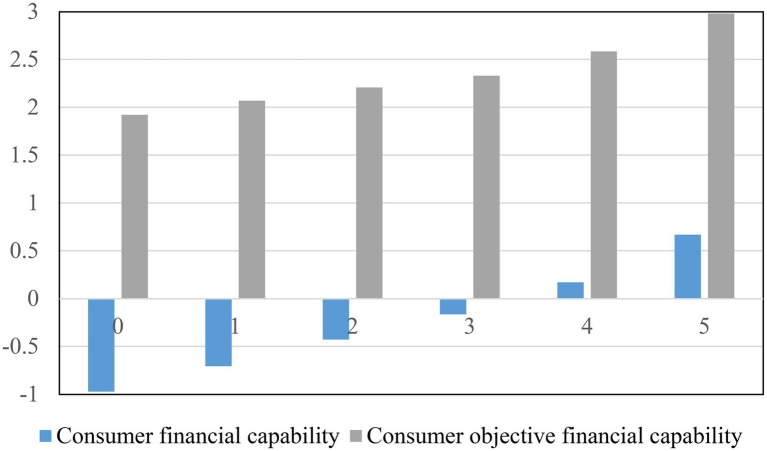
Consumer financial knowledge and financial capability.

According to the National Financial Capability Study (NFCS) data in 2012, 2015, and 2018, the mean values of financial knowledge are 3.465, 3.291, and 3.128, respectively. It implies that consumer financial knowledge was gradually declining in the recent three-wave surveys. Nevertheless, financial knowledge is positively associated with consumer (objective) financial capability (Figure 1). Hence, for adult consumers, informal financial education is important because it can improve their financial knowledge level. Besides, as early as the 1980s, there are many employers developing substantial financial education programs to provide employees with financial knowledge on financial decision-making behaviors and suggestions on individual retirement plans. Meanwhile, more evidence showed that informal financial education was developing at a rapid rate in the 1990s. However, little research shed light on informal financial education and its impact on consumer financial capability. Unlike prior studies, this study investigates the effects of informal financial education on consumer financial capability and further explores the mediating role of financial knowledge.

Consumer financial capability is a comprehensive measure of consumer financial knowledge, skills, values as well as attitudes, and it includes the ability to obtain financial products and services that are financially secure for themselves (Xiao and O’Neill, 2016). Meanwhile, consumer financial decision-making is a complex process that requires a lot of time and effort to search for and analyze the information needed for decision-making (Greenberg and Hershfield, 2019). Therefore, financial knowledge plays an essential role in the process of information screening and analysis (Kadoya and Khan, 2020). Besides, as household finance has been in the spotlight in recent years, the limited participation of households in the financial market, the allocation of financial assets as well as their influencing factors are the core issues of household finance research (Campbell, 2006). The structure and income of household asset allocation are closely related to the levels of their financial capability. The enhancement of consumer financial capability enables consumers to deepen their understanding of financial issues in daily life and improve their level of financial decision-making. Therefore, the role of consumer financial capability in household asset allocation is becoming increasingly prominent. Besides, based on individual rational thinking, consumers make reasonable investment decisions, take risks, and enjoy benefits, to achieve a higher level of financial satisfaction. The research on informal financial education is of great significance in consumer financial capability, which is not only conducive to an in-depth understanding of the factors affecting consumer financial capability and providing a reference basis for the formulation of policies by relevant departments, but also crucial to improving consumer financial knowledge through financial education, optimizing consumption and investment strategies, to enhance consumer financial wellbeing.

This study contributes to the literature of consumer financial capability by exploring the role of informal financial education, a vital policy variable that may be utilized to improve consumer financial decision-making. This study also contributes to the literature of the mediating effects of financial knowledge on the relationships between informal financial education and consumer financial capability by categorizing financial knowledge into objective and subjective financial knowledge, a unique perspective to examine the mediation of financial knowledge. Therefore, the findings of this study can be informative for consumer policymakers and educators to formulate informal financial education programs to help enhance consumer financial capability.

The purpose of this study is to explore the associations between informal financial education and consumer financial capability. The remainder of this paper is organized as follows. *Literature review and hypotheses* reviews the literature in related fields and develops the hypotheses. The sample data and measurements of variables are introduced in *Methodology*. *Empirical results* presents descriptive statistics and empirical results, and further addresses the mediating role of financial knowledge. *Conclusions, implications and limitations* concludes and puts forward implications and limitations.

## Literature review and hypotheses

### Previous research on informal financial education

Due to the increasing complexity of financial decision-making, the impact of financial education, especially informal financial education, on financial capability is inconclusive. On the one hand, financial education may play an insignificant role in improving consumer financial capability. For instance, Mandell and Klein (2009) argued that financial education has not significantly optimized financial decisions. Furthermore, outdated financial education is easy to make consumers fall into overconfidence, leading to undesirable investment decisions (Brown et al., 2016). According to Beckker et al. (2021), even though financial education may improve consumer financial capability, financial education does not automatically result in better financial decisions and financial outcomes. On the other hand, more researchers believe that financial education has a positive impact on financial capability. Previous studies suggest that financial education positively contributes to consumer financial capability, optimizes investment decisions, and enhances financial wellbeing (Alsemgeest, 2015; Xiao and O’Neill, 2016). Meanwhile, Wagner and Walstad (2019) suggested that financial education may help consumers make more optimal financial decisions and change inappropriate financial behaviors, which can have a positive impact on the financial wellbeing of households.

The acquisition of financial knowledge can be from formal financial education and informal financial education. Accordingly, formal financial education can be defined as learning knowledge and skills about finance in schools (Moscarola and Kalwij, 2021), Informal financial education is considered that consumer’s acquisition of financial knowledge depends on investment experience, social information, family environment impact, and the understanding of financial information in the workplace (Prawitz and Cohart, 2014). For consumers, to acquire new financial knowledge, the learning of financial knowledge takes place not only in schools or universities but also in communities or workplaces. Common financial education is carried out more in workplaces or communities, through the virtual space provided by financial education institutions or mobile apps. Meanwhile, modern companies increasingly provide training opportunities for employees to improve their financial knowledge through organized but informal training (Rudeloff, 2019). However, there is little evidence showing that formal financial education has played a more important role in enhancing consumer financial capability than informal financial education received from workplaces or communities rather than schools or universities (Moscarola and Kalwij, 2021).

### Prior studies on consumer financial capability

The definition of financial capability refers to the ability to use appropriate financial knowledge to carry out desirable financial decisions to obtain adequate financial satisfaction (Hoelzl and Kapteyn, 2011). Financial capability is determined by the level of financial knowledge and financial behaviors that can produce higher financial returns (Xiao et al., 2014). Therefore, financial knowledge and financial behavior are closely related to financial capability which is of great significance to the accumulation of wealth for consumers.

The studies on the influencing factors of consumer financial capability primarily focus on the determinants of financial risky asset holdings, and different explanations are given in the literature. First, consumer demographic variables such as age (Zhang et al., 2018), gender (Jacobsen et al., 2014), education levels (Bogan, 2015), and wealth holdings (Brown et al., 2021) all have an impact on household financial risky asset allocation. More specifically, household risky asset holdings increase with age (Zhang et al., 2018), and male investors are more likely to hold more risky assets than women (Jacobsen et al., 2014). Furthermore, increasing income levels and accumulating individual assets make consumers more likely to pay the inherent costs of participating in the financial market. Secondly, the subjective factors affect consumer financial risky assets holdings and financial capability. Specifically, higher levels of financial knowledge will drive consumers’ participation in financial markets and increase consumers’ holding of risky assets, especially equity assets (Giofré, 2017). Furthermore, consumers with higher financial planning ability and a higher level of risk awareness prefer to invest in financial markets and hold a larger proportion of risky assets (Nguyen et al., 2016). In addition, consumer social interaction and trust, and personal characteristics all have a significant impact on their holding of risky assets. More specifically, Meier and Sprenger (2013) indicated that individuals who prefer to acquire financial information may have embarked on the path to better financial outcomes. In previous studies, the measurement of consumer financial capability combines objective and subjective measurements of financial knowledge and financial behaviors (Xiao et al., 2015). The advantage of this method is that it is a more comprehensive measurement of financial capability, including both financial knowledge and consumer financial behaviors.

Financial education in the broad sense is considered as a kind of cultural and financial knowledge popularization. As one aspect of financial education, the impact of informal financial education on consumer financial capability has not been unanimously concluded. In addition to the financial education provided in schools or universities, workplaces or communities are important places for consumers to receive informal financial education. However, several studies suggest that informal financial education does not have a significantly positive impact on consumer financial capability. According to Hastings et al. (2013), although individuals’ experience has the potential to be an important learning method in the financial market, there are many pivotal financial decision-making behaviors within the consumer life cycle, such as asset investment and purchasing pension products for retirement, debt mortgage or educational investment, and the like. These financial behaviors are infrequently made and the results are delayed, and they will be subject to a large random impact. In this case, learning while doing may not be the most efficient way to acquire enough financial knowledge and make desirable financial decisions. Nevertheless, more studies have shown that informal financial education received in workplaces or communities plays a very crucial role in their financial wellbeing. According to Beck (2010), financial education provided by employers positively drops off consumers’ financial anxiety about future retirement, and helps consumers better plan for the future and maintain job stability. Furthermore, Hira and Loibl (2016) suggested that consumers are highly interested in comprehensive financial knowledge training provided by companies, and concluded that financial education in the workplace has the potential to enhance consumer financial capability. Thus, the following hypothesis is developed as

*H1*: Informal financial education is positively associated with consumer financial capability.

### Previous studies of financial knowledge on the nexus between information financial education and financial capability

Consumer financial education may affect financial behaviors and capability by improving their financial knowledge. Financial knowledge measures the extent of understanding basic financial concepts and also is considered as the ability and confidence to manage personal finances through appropriate, short-term decision-making and sound, long-range financial planning, when mindful of life events and changing economic conditions (Remund, 2017). Besides, previous research has shown that financial knowledge may serve as a mediator between financial education and financial capability in different income classes (Son and Park, 2018). Furthermore, household members with better counting ability and financial knowledge are more likely to actively participate in financial markets and hold risky assets (Lu et al., 2021). In particular, the lack of financial knowledge makes it impossible for a consumer to optimize their financial wellbeing, especially in high-risk financial situations, or in a financial environment of increased competitive pressure to improve market efficiency (Hastings et al., 2013). Besides, Rooij et al. (2011) used the data of the Dutch Central Bank Household Survey and indicated that most respondents only have basic financial knowledge and do not have slightly more professional financial knowledge, which has greatly restricted the capability of these consumers to make financial decisions.

Forms of financial education provided by employers may affect a large number of employees in their financial decision-making behaviors, including participation in the financial market, the portfolio of household risky assets, and the overall savings rate (Garrett, 2003). Bernheim and Garrett (2009) indicated that employees’ enthusiasm for voluntary participation in savings will increase and contributions may be more active when employers provide retirement seminars. As the financial market continues to mature, financial products have shown the characteristics of diversification and complexity. The fast-growing financial market requires consumers to decide their asset allocation and be responsible for their financial behaviors, which requires consumers to have higher financial knowledge. Financial knowledge and financial skills have received increasing attention as essential factors in making desirable financial decisions (Hastings et al., 2013). Furthermore, financial knowledge has played a pivotal role in improving consumer financial capability. Prior studies have suggested that consumers with a higher level of financial knowledge have a better outcome in financial decision-making about their asset allocation and retirement planning for the future (Lusardi and Mitchell, 2007b). However, financial knowledge is low even in developed economies where financial markets are well-developed (Lusardi, 2019). Simultaneously, previous studies have implied that fewer than one-third of the global population has an intimate knowledge of the fundamental concepts of daily financial decision-making (Lusardi and Mitchell, 2011). Moreover, Chu et al. (2017) argued that consumers with low financial knowledge have the potential to make undesirable financial decisions in asset allocation, insurance participation, financial planning, and retirement planning. In addition. Therefore, it is critical to improving consumer financial knowledge since these poor financial decisions may mitigate consumer financial wellbeing.

Consumer financial knowledge can be divided into subjective financial knowledge and objective financial knowledge. The measurement of subjective financial knowledge reflects the consumers’ overall evaluation of financial capability, and the willingness to answer questions about future return expectations on household assets is conceptually related to financial knowledge (Paiella, 2016). Consumer subjective financial knowledge measures their mastery of basic financial knowledge, financial concepts, and the capability to use and manage funds to effectively allocate financial resources to achieve lifelong financial security (Huston, 2010). Specifically, consumer overestimation of financial capability may lead to undesirable financial behaviors. According to Kramer (2016), overconfident retail investors are reluctant to seek professional financial advice in planning insurance or allocating household assets. Besides, through investigating the relationships between subjective financial knowledge and trading behaviors, Anthony et al. (2018) indicated that subjective financial knowledge helps explain the horizontal differences in the behaviors of retail investors, and the investors who report a higher level of financial knowledge seem to have a higher yield. Consumer objective financial knowledge measures individuals’ understanding of basic financial knowledge (Lind et al., 2020). Lusardi and Mitchell (2007a) revealed that among elderly consumers in the United States, those who acquire more financial knowledge tend to have a greater probability of making budgets and are more likely to invest in complex financial products. Similarly, consumers with lower objective financial knowledge find it hard to appropriately choose financial products and services for their financial situations and avoid abusive practices by relying on their financial knowledge (Agnew and Szykman, 2005). In addition, over-cognition of financial knowledge is one of the reasons for suboptimal financial outcomes (Balasubramnian and Sargent, 2020). As a result, the following hypotheses are developed:

*H2*: Financial knowledge, incorporating objective and subjective financial knowledge, mediates the associations between informal financial education and consumer financial capability.

## Methodology

### Data

The data of this study come from the 2012, 2015, and 2018 NFCS in the United States, and it is available on the website of the Financial Industry Regulatory Authority (FINRA) Investor Education Foundation. The NFCS aims to comprehensively analyze the financial capacity of nations through a rich and interrelated set of perceptions, attitudes, experiences, and behaviors. The FINRA Investor Education Foundation commissioned the first national study in 2009. So far, four rounds have been carried out in 2009, 2012, 2015, and 2018, respectively. The survey content covers the balance of payments, planning ahead, financial product management, financial knowledge as well as decision. Through the analysis of the survey, this study can count and analyze the financial knowledge of American consumers, find out the key indicators of financial capability, and identify the factors that affect relevant indicators. For this study, some observations were dropped for respondents who answered “Do not know” or “Prefer not to say,” and to confirm that consumers’ responses are reliable, samples with household heads aged under 25 and over 65 are removed. Thus, the sample size of this study is 16,736.

### Variables

In this study, the dependent variable is consumer financial capability, which is categorized as objective financial capability and subjective financial capability. Subjective financial capability is measured on a 7-point scale. Respondents are asked “How strongly do you agree or disagree with the following statements? - I am good at dealing with day-to-day financial matters, such as checking accounts, credit, debit cards, and tracking expenses” 1 indicates “strongly disagree” and 7 stands for “strongly agree.” Objective financial capability is measured by a 5-point scale. The respondents were asked five questions related to income and expenditure, debt repayment, emergency savings, child education savings as well as saving for retirement, if they performed any of these behaviors, the variable is encoded 1, otherwise 0. Moreover, financial capability is the sum of Z-scores of objective financial capability and subjective financial capability. The independent variable of informal financial education is measured by the question worded “Did you receive financial education from an employer?” If they have received any financial education from the employers, the variable is encoded 1, otherwise 0.

The measurement of financial knowledge is divided into subjective financial knowledge and objective financial knowledge. In addition, financial knowledge is the sum of Z-scores of objective financial knowledge and subjective financial knowledge. In detail, subjective financial knowledge is measured on a 7-point scale. Respondents were asked, “On a scale from 1 to 7, where 1 means very low and 7 means very high, how would you assess your overall financial knowledge?” Meanwhile, objective financial knowledge is measured on a 6-point scale. The respondents were asked six questions about compound interest, inflation, bond price, mortgage interest, repayment interest, and risk diversification, the respondents would get one point if they answer each question correctly with total points of six. There are some demographic variables included in the study as control variables, such as age, gender, education, marital status, race, and the like. To address the associations between informal financial education and consumer financial capability, the control variables, such as risk attitude, participation in the financial markets, credit record rating, annual income, number of financially dependent children, and subjective math capability, are incorporated as well. The specifications of all variables are presented in Table 1.

**Table 1:** Specifications of variables.

Variable type	Variable lable	Variables	Measurement	Attribute
Dependent variables	*fcap*	Financial capability	A sum of Z-scores of objective financial capability and subjective financial capability	
	*sfcap*	Subjective financial capability	The sum of five questions related to income and expenditure, debt repayment, saving for emergencies, saving for children’s education, and saving for retirement	For each question: 1 = Ture, and 0 = False
	*ofcap*	Objective financial capability	“How strongly do you agree or disagree with the following statements? - I am good at dealing with day-to-day financial matters, such as checking accounts, credit, and debit cards, and tracking expenses.”	From 1 = Strongly disagree to 7 = Strongly agree
Independent variables	*ife*	Informal financial education	“Did you receive financial education from an employer?”	1 = Yes, and 0 = No
Control variables	*male*	Gender		1 = Male, and 0 = Female	
	*age1*	Age 18–24		1 = Yes, and 0 = No	
	*age2*	Age 25–34		1 = Yes, and 0 = No	
	*age3*	Age 35–44		1 = Yes, and 0 = No	
	*age4*	Age 45–54		1 = Yes, and 0 = No	
	*age5*	Age 55–64		1 = Yes, and 0 = No	
	*age6*	Age 65 or above		1 = Yes, and 0 = No	
	*edu1*	High school or below		1 = Yes, and 0 = No	
	*edu2*	Some colleges to bachelor’s degrees		1 = Yes, and 0 = No	
	*edu3*	Postgraduate degrees or above		1 = Yes, and 0 = No	
	*white*	White		1 = White, and 0 = Non-white	
	*married*	Marital status		1 = Married, 0 = Otherwise	
	*income*	Annual income	“What is your (household’s) approximate annual income, including wages, tips, investment income, public assistance, income from retirement plans, etc.”	1 = Less than $15,000, 2 = $15,000 to $25,000, 3 = $ 25,000 to $35,000, 4 = $35,000 to $50,000, 5 = $50,000 to $75,000, 6 = $75,000 to $100,000, 7 = $100,000 to 150,000, 8 = $150,000 or more	
	*mathcap*	Math capability	“How strongly do you agree or disagree with the following statements? - I am pretty good at math.”	From 0 (low) to 7 (high)	
	*risk*	Risk attitude	“When thinking of your financial investments, how willing are you to take risks?”	From 0 (low) to 10 (high)	
	*child*	Number of financially dependent children	“How many children do you have who are financially dependent on you (or your spouse/partner)?”	From 0 to 4 (or more)	
	*credit*	Credit record rating	“How would you rate your current credit record?”	From 0 (low) to 5 (high)	
	*pfm*	Participating in the financial markets	“Not including retirement accounts, do you [does your household] have any investments in stocks, bonds, mutual funds, or other securities?”	1 = Yes, and 0 = No
Mediating variables	*fk*	Financial knowledge	A sum of Z-scores of objective and subjective financial knowledge	
	*sfk*	Subjective financial knowledge	“How would you assess your overall financial knowledge?”	From 0 (low) to 7 (high)
	*ofk*	Objective financial knowledge	The sum of six questions about compound interest, inflation, bond price and mortgage interest, repayment interest, and risk diversification	For each question: 1 = Ture, and 0 = False

The content is arranged by the authors.

### Data analysis

In this study, the unweighted samples are utilized to produce the results of descriptive statistics, correlations, and multivariate regressions. Since the dependent variables of objective and subjective financial capability are not continuous but ordered, the method of ordered probit regression is employed. As for the dependent variable of consumer financial capability, this study follows the approaches made by Xiao et al. (2014), and it is calculated by a sum of Z-scores of objective and subjective financial capability, which is continuous. Hence, the approach of ordinary least squares (OLS) regression is utilized. To check the robustness, the alternative methods of ordered logit regression and feasible weighted least squares (FWLS) are used. In addition, the approaches of the instrumental variable and 2SLS are utilized to mitigate the bias caused by endogeneity. Furthermore, the Sobel test is performed to verify the adequacy of financial knowledge as a mediator.

## Empirical results

### Results of descriptive statistics

The results of the descriptive statistics are displayed in Table 2. In detail, consumer age is defined as categorized variables specific to aged 18–24, 25–34, 35–44, 45–54, 55–64, and 65 or above, respectively. Similarly, consumer education levels are, respectively, indicated as high school or below, some colleges to bachelor’s degrees, and post-graduate degrees or above. For the dependent variable of financial capability, it is the sum of Z-scores of subjective financial capability and objective financial capability, thus the mean of financial capability is 0. The mean score of subjective financial capability is 5.977 out of 7, which implies most consumers consider they have a high financial capability. However, the mean score of objective financial capability is 2.493 out of 5, which indicates that fewer than half of consumers have a high level of objective financial capability. Through the measurement of consumer subjective and objective financial capability, the results suggest that consumers tend to overestimate their financial capability. Meanwhile, the same happens in the measurement of financial knowledge, the mean score of objective and subjective financial knowledge are 3.291 out of 5 and 5.353 out of 7, which also indicates people tend to exaggerate their financial knowledge. For the independent variable of informal financial education, the mean score is 0.390, which indicates that fewer than half of consumers have received financial education from their employers.

**Table 2:** Descriptive statistics.

Variable	Obs	Mean	Std. dev.	Min	Max
*fcap*	16,736	0.000	1.593	−6.009	2.596
*sfcap*	16,736	5.977	1.448	0	7
*ofcap*	16,736	2.493	1.327	0	5
*fk*	16,736	0.000	1.562	−6.878	2.420
*ofk*	16,736	3.291	1.397	0	5
*sfk*	16,736	5.535	1.224	0	7
*ife*	16,736	0.390	0.488	0	1
*risk*	16,736	5.468	2.663	0	10
*pfm*	16,736	0.434	0.496	0	1
*credit*	16,736	2.648	2.130	0	5
*income*	16,736	4.734	2.095	1	8
*male*	16,736	0.504	0.500	0	1
*age1*	16,736	0.147	0.354	0	1
*age2*	16,736	0.194	0.395	0	1
*age3*	16,736	0.163	0.370	0	1
*age4*	16,736	0.179	0.383	0	1
*age5*	16,736	0.159	0.366	0	1
*age6*	16,736	0.158	0.365	0	1
*edu1*	16,736	0.103	0.303	0	1
*edu2*	16,736	0.470	0.499	0	1
*edu3*	16,736	0.115	0.319	0	1
*married*	16,736	0.550	0.498	0	1
*mathcap*	16,736	5.930	1.505	0	7
*white*	16,736	0.697	0.459	0	1
*child*	16,736	0.745	1.091	0	4

The results of descriptive statistics are from the data of the 2012, 2015, and 2018 National Financial Capability Study.

### Results of correlation analysis

Table 3 represents the correlations between the variables of informal financial education, financial capability, and the like. Consumer subjective and objective financial capability are positively correlated with informal financial education, and the correlation coefficients are 0.134 and 0.188 at the significance level of 1%, respectively. More specifically, informal financial education has a significant positive impact on financial capability, with a correlation coefficient of 0.202 at the 1% significance level. Since financial knowledge is constructed through two variables, subjective financial knowledge, and objective financial knowledge, the correlation coefficient is high and significant. As for the other control variables, most of the correlations are as expected, which is also verified by Xiao et al. (2014). For example, participation in financial markets, credit history ratings, risk attitudes, and income are positively associated with consumer financial capability.

**Table 3:** Results of correlation analysis.

	*fcap*	*sfcap*	*ofcap*	*fk*	*ofk*	*sfk*	*ife*	*risk*	*pfm*	*credit*	*income*
*fcap*	1										
*sfcap*	0.796***	1									
*ofcap*	0.796***	0.269***	1								
*fk*	0.467***	0.408***	0.335***	1							
*ofk*	0.294***	0.234***	0.234***	0.781***	1						
*sfk*	0.435***	0.404***	0.289***	0.781***	0.219***	1					
*ife*	0.202***	0.134***	0.188***	0.213***	0.135***	0.197***	1				
*risk*	0.240***	0.0875***	0.294***	0.208***	0.061***	0.264***	0.120***	1			
*pfm*	0.377***	0.180 ***	0.420***	0.313***	0.238***	0.252***	0.206***	0.323***	1		
*credit*	0.229***	0.135***	0.230***	0.110***	0.060***	0.112***	0.042***	0.133***	0.128***	1	
*income*	0.438***	0.215***	0.482***	0.365***	0.313***	0.257***	0.247***	0.269***	0.397***	0.193***	1

The sample size is 16,736, and the unweighted sample is employed. Besides, ***, **, and * denote statistical significance at the 1%, 5%, and 10% levels, respectively.

### Results of benchmark estimations

Table 4 represents the benchmark regression results of informal financial education on consumer financial capability. In Column (1), only the control variables are included, and most of their coefficients are as expected. In Columns (2) to (6), the independent variable of informal financial education is incorporated simultaneously. In Columns (1), (2), (3), and (6), the approach of OLS regression is utilized. In addition, in Columns (4) and (5), the ordered probit regressions are further performed. To eliminate the influence of regional heterogeneity on the estimation results, the state dummy variables are controlled in all estimates. In Columns (4) and (5), consumers who have received informal financial education have a higher subjective and objective financial capability. To further investigate the relationship between informal financial education and consumer financial capability, Column (6) reports the estimation results that informal financial education positively contributes to consumer financial capability, which is aligned with *H1*. In addition, consumers with higher risk tolerance and participation in financial markets frequently, as well as high credit ratings, have a higher level of financial capability. The results are coherent with Chen et al. (2020).

**Table 4:** Results of regressions on consumer financial capability.

Variables	(1)	(2)	(3)	(4)	(5)	(6)
	*sfcap*	*sfcap*	*ofcap*	*sfcap*	*ofcap*	*fcap*
*ife*		0.135***	0.097***	0.179***	0.089***	0.167***
		(0.020)	(0.021)	(0.022)	(0.020)	(0.022)
*risk*	0.002	0.001	0.047***	0.002	0.045***	0.036***
	(0.004)	(0.004)	(0.003)	(0.004)	(0.003)	(0.004)
*pfm*	0.200***	0.185***	0.617***	0.228***	0.584***	0.593***
	(0.023)	(0.024)	(0.021)	(0.026)	(0.019)	(0.023)
*credit*	0.081***	0.080***	0.120***	0.083***	0.113***	0.146***
	(0.007)	(0.007)	(0.006)	(0.006)	(0.005)	(0.007)
*income*	0.051***	0.046***	0.187***	0.049***	0.176***	0.173***
	(0.005)	(0.005)	(0.006)	(0.005)	(0.006)	(0.006)
*male*	−0.177***	−0.178***	0.063***	−0.174***	0.061***	−0.075***
	(0.021)	(0.021)	(0.018)	(0.020)	(0.018)	(0.020)
*age2*	−0.022	−0.018	0.109***	−0.098***	0.110***	0.069**
	(0.030)	(0.030)	(0.027)	(0.029)	(0.026)	(0.029)
*age3*	0.022	0.023	0.105***	−0.056*	0.115***	0.095**
	(0.035)	(0.035)	(0.030)	(0.032)	(0.029)	(0.037)
*age4*	0.080***	0.079***	0.134***	0.026	0.150***	0.155***
	(0.024)	(0.024)	(0.029)	(0.025)	(0.027)	(0.031)
*age5*	0.191***	0.179***	0.123***	0.203***	0.128***	0.217***
	(0.026)	(0.026)	(0.026)	(0.027)	(0.025)	(0.026)
*edu2*	−0.173***	−0.168***	−0.248***	−0.208***	−0.234***	−0.303***
	(0.032)	(0.031)	(0.027)	(0.025)	(0.026)	(0.034)
*edu3*	−0.176***	−0.169***	−0.255***	−0.210***	−0.236***	−0.309***
	(0.040)	(0.040)	(0.032)	(0.038)	(0.032)	(0.040)
*married*	0.097***	0.094***	0.041*	0.098***	0.036*	0.096***
	(0.026)	(0.026)	(0.022)	(0.025)	(0.021)	(0.027)
*mathcap*	0.488***	0.486***	0.062***	0.385***	0.059***	0.382***
	(0.009)	(0.009)	(0.007)	(0.008)	(0.006)	(0.009)
*white*	0.074**	0.078***	−0.044*	0.079***	−0.043*	0.020
	(0.028)	(0.027)	(0.026)	(0.025)	(0.026)	(0.030)
*child*	−0.081***	−0.081***	0.065***	−0.080***	0.076***	−0.006
	(0.011)	(0.011)	(0.012)	(0.010)	(0.012)	(0.013)
*constant*	2.610***	2.605***	0.200***			−4.058***
	(0.057)	(0.057)	(0.048)			(0.059)
State fixed effect	Yes	Yes	Yes	Yes	Yes	Yes
Observations	167,36	16,736	16,736	16,736	16,736	16,736
Adjusted *R*^2^	0.328	0.330	0.338			0.403
Pseudo *R*^2^				0.128	0.121	

Reference groups are high school or below, age 18–24, and age 65 or above. The unweighted samples are utilized. In addition, ***, **, and * represent 1%, 5%, and 10% significance levels, respectively, and the data in parentheses are robust standard errors.

### Robustness, endogeneity, and heterogeneity check

To verify the robustness of the estimates, a comprehensive check is conducted. Firstly, an alternative approach of the ordered logit regression is utilized to perform re-estimates. The results are displayed in Columns (1) and (2) in Table 5. Meanwhile, Column (3) is estimated by the approach of the FWLS method. Secondly, this study also excludes samples with an annual income of less than $15,000 and more than $150,000, which is positive to eliminate the estimation bias caused by outliers. Accordingly, the results are reported in Columns (4) in Table 5. In terms of the re-estimation results, informal financial education is still positively associated with consumer financial capability. Meanwhile, for consumer subjective and objective financial capability, the results keep unchanged. In Column (4), after dropping the outliers of annual income less than $15,000 and more than $150,000, informal financial education is still statistically positive to consumer financial capability. Thus, the results of the robustness check endorse *H1* as well.

**Table 5:** Results of robustness check.

Variables	(1)	(2)	(3)	(4)	(5)
	*sfcap*	*ofcap*	*fcap*	*fcap*	*fcap*
*ife*	0.301***	0.144***	0.155***	0.176***	5.343***
	(0.037)	(0.035)	(0.020)	(0.025)	(0.607)
*risk*	−0.003	0.076***	0.036***	0.033***	0.004
	(0.007)	(0.006)	(0.004)	(0.005)	(0.006)
*pfm*	0.380***	1.016***	0.564***	0.582***	0.027
	(0.044)	(0.036)	(0.023)	(0.024)	(0.072)
*credit*	0.146***	0.194***	0.138***	0.162***	0.129***
	(0.011)	(0.010)	(0.007)	(0.007)	(0.008)
*income*	0.082***	0.310***	0.172***	0.178***	−0.032
	(0.008)	(0.010)	(0.007)	(0.007)	(0.025)
*male*	−0.320***	0.104***	−0.040**	−0.053**	−0.126***
	(0.032)	(0.031)	(0.017)	(0.021)	(0.019)
*age2*	−0.158***	0.187***	0.052*	0.056	0.191***
	(0.050)	(0.046)	(0.029)	(0.035)	(0.027)
*age3*	−0.096*	0.206***	0.099***	0.066	0.131***
	(0.053)	(0.052)	(0.035)	(0.042)	(0.038)
*age4*	0.035	0.279***	0.165***	0.134***	0.109***
	(0.042)	(0.048)	(0.030)	(0.035)	(0.031)
*age5*	0.335***	0.239***	0.205***	0.214***	−0.207***
	(0.047)	(0.044)	(0.023)	(0.029)	(0.058)
*edu2*	−0.371***	−0.398***	−0.330***	−0.356***	−0.119***
	(0.044)	(0.048)	(0.031)	(0.032)	(0.043)
*edu3*	−0.392***	−0.409***	−0.341***	−0.348***	−0.035
	(0.064)	(0.056)	(0.037)	(0.044)	(0.055)
*married*	0.134***	0.057	0.086***	0.081***	−0.037
	(0.045)	(0.036)	(0.025)	(0.028)	(0.027)
*mathcap*	0.743***	0.103***	0.384***	0.383***	0.305***
	(0.015)	(0.012)	(0.008)	(0.011)	(0.013)
*white*	0.126***	−0.075*	0.049*	0.030	0.177***
	(0.043)	(0.044)	(0.027)	(0.033)	(0.032)
*child*	−0.126***	0.119***	0.033**	−0.008	0.002
	(0.017)	(0.021)	(0.013)	(0.014)	(0.012)
*constant*			−3.882***	−3.973***	−4.257***
			(0.051)	(0.069)	(0.063)
State fixed effect	Yes	Yes	Yes	Yes	Yes
Observations	16,736	16,736	16,736	13,586	16,736
Adjusted *R*^2^			0.377	0.357	0.404
Pseudo *R*^2^	0.134	0.122			

The unweighted samples are utilized. In addition, ***, **, and * represent 1%, 5%, and 10% significance levels, respectively, and the data in parentheses are robust standard errors. Reference groups are high school or below, age 18–24, and age 65 or above.

This study also recognizes that there may be endogenous problems because the causal relationship between informal financial education and consumer financial capability cannot be determined only by the coefficient, and they may also face two-way causal problems. Therefore, the potential endogeneity of informal financial education must be approached with caution. The methods of instrumental variable (IV) and 2SLS are used in this study to eliminate the estimation bias caused by endogeneity. In this study, the variable indicating retirement plans provided by consumer companies, and consumer spouse’s companies, serves as an instrumental variable. The employers’ retirement plans are parts of the informal financial education and they are almost exogenous to consumer financial capability. Besides, the study shows that the value of F in the first stage regression is 25.38, greater than 10, which indicates that it is not a weak instrumental variable. In Table 5, Column (5) exhibits the results of the endogeneity test, the coefficient of informal financial education is statistically positive, which implies that informal financial education positively and significantly contributes to consumer financial capability. Therefore, the results of the endogeneity check endorse *H1* as well.

To address the heterogeneous effects of the region and age on affecting the impact of informal financial education on consumer financial capability, the study performs OLS regressions in the Northeast, Midwest, South, and West of the United States, the sample group aged 44 or below and the sample group aged 45 or above. Table 6 displays the results of the heterogeneity check. The estimated sample groups from Columns (1) to (6) are the northeast of the United States, the Midwest of the United States, the south of the United States, the west of the United States, the sample group aged 44 or below, and the sample group aged 45 or above, respectively. The impact of informal financial education on consumer financial capability has little heterogeneity in different regions. Simultaneously, the estimation results for both sample groups aged 44 or below as well as that aged 45 or above are positive to enhance consumer financial capability. Thus, the results are also coherent with *H1*.

**Table 6:** Results of the heterogeneous roles of region and age.

Variables	(1)	(2)	(3)	(4)	(5)	(6)
	*fcap*	*fcap*	*fcap*	*fcap*	*fcap*	*fcap*
*ife*	0.167***	0.165**	0.165***	0.157***	0.206***	0.106***
	(0.044)	(0.063)	(0.033)	(0.043)	(0.030)	(0.027)
*risk*	0.033**	0.022**	0.038***	0.047***	0.057***	0.022***
	(0.011)	(0.007)	(0.009)	(0.007)	(0.007)	(0.005)
*pfm*	0.601***	0.619***	0.619***	0.533***	0.618***	0.538***
	(0.058)	(0.059)	(0.033)	(0.044)	(0.037)	(0.029)
*credit*	0.175***	0.138***	0.132***	0.152***	0.138***	0.152***
	(0.018)	(0.012)	(0.013)	(0.013)	(0.011)	(0.011)
*income*	0.155***	0.178***	0.185***	0.168***	0.164***	0.193***
	(0.015)	(0.010)	(0.013)	(0.012)	(0.008)	(0.009)
*male*	−0.105	−0.060	−0.073**	−0.069	−0.098**	−0.047*
	(0.060)	(0.043)	(0.027)	(0.043)	(0.039)	(0.024)
*age2*	0.122	0.038	0.052	0.067*		
	(0.107)	(0.047)	(0.056)	(0.035)		
*age3*	0.188	0.104	0.109*	0.023		
	(0.121)	(0.058)	(0.057)	(0.078)		
*age4*	0.146	0.203***	0.136*	0.133***		
	(0.081)	(0.064)	(0.070)	(0.040)		
*age5*	0.138**	0.311***	0.153***	0.256***		
	(0.057)	(0.048)	(0.052)	(0.040)		
*edu2*	−0.438***	−0.281***	−0.238***	−0.330***	−0.224***	−0.401***
	(0.100)	(0.046)	(0.054)	(0.073)	(0.049)	(0.054)
*edu3*	−0.431***	−0.180**	−0.265***	−0.401***	−0.218***	−0.419***
	(0.103)	(0.080)	(0.052)	(0.086)	(0.074)	(0.058)
*married*	0.063	0.083	0.116**	0.110*	0.259***	−0.095**
	(0.075)	(0.062)	(0.042)	(0.051)	(0.039)	(0.035)
*mathcap*	0.371***	0.378***	0.385***	0.388***	0.351***	0.412***
	(0.020)	(0.013)	(0.017)	(0.020)	(0.012)	(0.013)
*white*	−0.009	−0.004	−0.061	0.165***	−0.004	−0.002
	(0.091)	(0.064)	(0.041)	(0.052)	(0.036)	(0.036)
*child*	−0.038	0.005	0.010	−0.013	−0.016	0.019
	(0.039)	(0.023)	(0.025)	(0.019)	(0.015)	(0.016)
*constant*	−3.767***	−3.822***	−3.872***	−4.036***	−3.609***	−3.627***
	(0.153)	(0.072)	(0.095)	(0.118)	(0.082)	(0.080)
State fixed effect	Yes	Yes	Yes	Yes	Yes	Yes
Observations	2,758	3,888	5,366	4,724	8,427	8,309
Adjusted *R*^2^	0.408	0.400	0.409	0.395	0.371	0.410

Reference groups are high school or below, age 18–24, and age 65 or above. Moreover, ***, **, and * represent 1%, 5%, and 10% significance levels, respectively, and the data in parentheses are robust standard errors.

### Results of the mediating effects of financial knowledge

**Figure 2:**
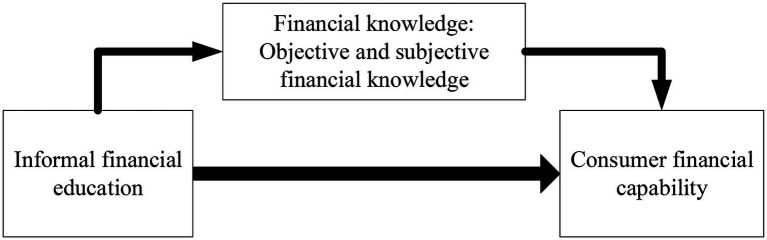
The mediating effects of financial knowledge.

To further address the influence channels between informal financial education and consumer financial capability, this study introduces financial knowledge as a mediator. In terms of Kaiser et al. (2022), financial knowledge is considered as a more comprehensive concept that includes not only basic financial knowledge but also an understanding of the financial market and the ability to make correct financial decisions in the face of financial market turbulence. Therefore, the study categorizes financial knowledge into subjective financial knowledge and objective financial knowledge, which is positive to more clearly investigate the influence channels between informal financial education and consumer financial capability. The mediation framework of financial knowledge is offered in Figure 2.

Since the variable of consumer financial capability is continuous, the approach of OLS regression is utilized to examine the mediating effects of financial knowledge. Table 7 shows the results of mediating effects of financial knowledge. In Columns (1) and (2), objective financial knowledge serves as a mediating variable, and subjective financial knowledge works as a mediating variable in Columns (3) and (4). Moreover, financial knowledge works as the mediating variable in Columns (5) and (6).

**Table 7:** Results of the mediating effects of financial knowledge.

Variables	(1)	(2)	(3)	(4)	(5)	(6)
	*ofk*	*fcap*	*sfk*	*fcap*	*fk*	*fcap*
*ofk*		0.079***				
		(0.008)				
*sfk*				0.256***		
				(0.010)		
*fk*						0.200***
						(0.009)
*ife*	0.094***	0.159***	0.247***	0.103***	0.269***	0.113***
	(0.020)	(0.022)	(0.020)	(0.020)	(0.021)	(0.020)
*risk*	−0.023***	0.038***	0.073***	0.018***	0.044***	0.028***
	(0.005)	(0.004)	(0.005)	(0.004)	(0.005)	(0.004)
*pfm*	0.285***	0.571***	0.214***	0.538***	0.379***	0.517***
	(0.026)	(0.023)	(0.018)	(0.023)	(0.024)	(0.023)
*credit*	−0.044***	0.149***	0.043***	0.135***	0.004	0.145***
	(0.006)	(0.007)	(0.007)	(0.007)	(0.007)	(0.007)
*income*	0.114***	0.164***	0.042***	0.162***	0.116***	0.149***
	(0.007)	(0.007)	(0.006)	(0.006)	(0.007)	(0.006)
*male*	0.359***	−0.104***	0.101***	−0.101***	0.340***	−0.143***
	(0.022)	(0.019)	(0.017)	(0.019)	(0.022)	(0.019)
*age2*	−0.334***	0.095***	−0.051**	0.082***	−0.281***	0.125***
	(0.030)	(0.029)	(0.024)	(0.028)	(0.031)	(0.028)
*age3*	−0.015	0.096**	−0.062*	0.111***	−0.061	0.108***
	(0.038)	(0.036)	(0.031)	(0.036)	(0.039)	(0.034)
*age4*	0.215***	0.138***	−0.071***	0.174***	0.096***	0.136***
	(0.032)	(0.031)	(0.031)	(0.032)	(0.036)	(0.031)
*age5*	0.260***	0.196***	0.088***	0.194***	0.257***	0.165***
	(0.030)	(0.026)	(0.027)	(0.024)	(0.035)	(0.024)
*edu2*	0.123***	−0.313***	−0.155***	−0.263***	−0.039	−0.295***
	(0.027)	(0.034)	(0.035)	(0.033)	(0.034)	(0.033)
*edu3*	0.432***	−0.343***	−0.105***	−0.282***	0.223***	−0.354***
	(0.051)	(0.039)	(0.039)	(0.040)	(0.046)	(0.038)
*married*	0.157***	0.084***	0.085***	0.074***	0.182***	0.060**
	(0.026)	(0.026)	(0.023)	(0.025)	(0.029)	(0.025)
*mathcap*	0.173***	0.369***	0.243***	0.320***	0.322***	0.318***
	(0.007)	(0.009)	(0.009)	(0.010)	(0.009)	(0.009)
*white*	0.372***	−0.009	0.030	0.013	0.291***	−0.038
	(0.032)	(0.029)	(0.025)	(0.028)	(0.036)	(0.028)
*child*	−0.122***	0.003	−0.013	−0.003	−0.099***	0.013
	(0.011)	(0.012)	(0.008)	(0.012)	(0.010)	(0.012)
*constant*	1.157***	−4.150***	3.221***	−4.882***	−3.419***	−3.374***
	(0.054)	(0.062)	(0.063)	(0.071)	(0.065)	(0.057)
State fixed effect	Yes	Yes	Yes	Yes	Yes	Yes
Observations	16,736	16,736	16,736	16,736	16,736	16,736
Adjusted *R*^2^	0.251	0.406	0.234	0.432	0.339	0.428

Reference groups are high school or below, age 18–24, and age 65 or above. Moreover, ***, **, and * represent 1%, 5%, and 10% significance levels, respectively, and the data in parentheses are robust standard errors.

The coefficients of informal financial education on consumer objective financial knowledge, subjective financial knowledge, and financial knowledge are significantly positive, and the coefficients of objective financial knowledge, subjective financial knowledge, and financial knowledge on consumer financial capability are also significantly positive. The regression results indicate that objective and subjective financial knowledge play mediating roles in the impact of informal financial education on consumer financial capability, and so does financial knowledge, which is aligned with *H2*. Besides, for the Sobel test of the mediating effects of objective financial knowledge, subjective financial knowledge, and financial knowledge, the *Z* values are 4.251, 12.55, and 11.56, respectively, all of which are at the significance level of 1%. Thus, the null hypothesis of the Sobel test was significantly rejected. In addition, after adding mediating variables, the coefficients of informal financial education have decreased, indicating that financial knowledge, incorporating objective and subjective financial knowledge, serves as partial mediators between informal financial education and consumer financial capability.

## Conclusions, implications and limitations

As income levels rise, increasing consumers will consider investing more in risky assets to accumulate wealth. Financial capability is an important source of driving power that accelerates economic development and plays a vital role in improving consumer financial wellbeing. Utilizing the data from the NFCS in 2012, 2015, and 2018, this study employs the approaches of OLS and ordered probit regressions to explore the impact of informal financial education on consumer financial capability. The results reveal that informal financial education has played a significant and positive role in enhancing consumer financial capability. Simultaneously, the mediating roles of financial knowledge, incorporating objective and subjective financial knowledge, are further investigated as well. The results suggest that financial knowledge partially mediates the associations between informal financial education and consumer financial capability.

Although consumers have accumulated investment experience in the process of continuously participating in the financial market, they will still face huge investment risks in the financial market if they are not well equipped with financial capability. The findings of this study suggest the importance of promoting informal financial education, which helps consumers make better risky asset portfolios and improve financial income to make household finance healthy. Therefore, informal financial education becomes more and more important. Besides, financial knowledge has been brought into sight in recent years. Meanwhile, developed economies such as the UK and the United States generally attach importance to financial education, regarding financial education as a national strategy. The empirical findings of this study are informative for policymakers and financial institutions to take effective measures to popularize financial education in workplaces, communities, and the like, which positively enhances consumer financial capability. First, effective measures are encouraged to be formulated to enhance financial education, not only integrating financial education into the national education system but also constructing additional programs of informal financial education for adult consumers. Besides, with the development of Internet finance, policymakers should pay more attention to Internet financial education, which is pivotal to consumer financial security. Second, general companies, as the main providers of informal financial education, should promote the financial education and training of employees to enhance their financial capability. Especially, companies can regularly invite well-known experts and scholars to offer financial courses, organize employees to learn financial knowledge, and then improve their financial capability, which is positive to enhance employees’ awareness of property protection, risk diversifications, and rational investments. Third, consumers are encouraged to actively receive informal financial education, improve their financial capability, and then enhance their financial wellbeing.

Limitations of this study should be acknowledged and outlined, which is positive to guide further research directions. First, although the research data comes from the NFCS in 2012, 2015, and 2018, it is still a cross-sectional one. To address the dynamic relationships between informal financial education and consumer financial capability, panel data should be utilized. Second, although consumer financial capability is measured from objective and subjective perspectives, other determinants may also matter. A more comprehensive measuring system of financial capability needs to be constructed in the future. Third, only the US data is used in this study. Data from other countries, especially from developing economies, should be incorporated, which is positive to produce more general results.

## Data availability statement

Publicly available datasets were analyzed in this study. This data can be found at: http://www.usfinancialcapability.org/downloads.php.

## Author contributions

FC and WW: conceptualization, writing—review and editing, supervision, project administration, funding acquisition. FC and XL: methodology, writing—original draft preparation. XL: resources, data collection. All authors have read and agreed to the published version of the manuscript.

## Funding

The authors acknowledge the financial support of the General Research Fund of The Academy of China Open Economy Studies at University of International Business and Economics (Grant No. 2022GK10) and Postgraduate Innovative Research Fund of University of International Business and Economics (Grant No. 202240).

## Conflict of interest

The authors declare that the research was conducted in the absence of any commercial or financial relationships that could be construed as a potential conflict of interest.

## Publisher’s note

All claims expressed in this article are solely those of the authors and do not necessarily represent those of their affiliated organizations, or those of the publisher, the editors and the reviewers. Any product that may be evaluated in this article, or claim that may be made by its manufacturer, is not guaranteed or endorsed by the publisher.
